# Hepatoprotective activity of *Lepidium sativum* seeds against D-galactosamine/lipopolysaccharide induced hepatotoxicity in animal model

**DOI:** 10.1186/s12906-016-1483-4

**Published:** 2016-12-03

**Authors:** Mohammad Raish, Ajaz Ahmad, Khalid M. Alkharfy, Syed Rizwan Ahamad, Kazi Mohsin, Fahad I. Al-Jenoobi, Abdullah M. Al-Mohizea, Mushtaq Ahmad Ansari

**Affiliations:** 1 Department of Pharmaceutics, College of Pharmacy, King Saud University, PO Box 2457, Riyadh, 11451 Saudi Arabia; 2 Clinical Pharmacy, King Saud University, Riyadh, 11451 Saudi Arabia; 3 Research Centre, King Saud University, Riyadh, 11451 Saudi Arabia; 4 Pharmacology College of Pharmacy, King Saud University, Riyadh, 11451 Saudi Arabia

**Keywords:** Hepatotoxicity, *Lepidium sativum*, D-galactosamine/lipopolysaccharide, Cytokines

## Abstract

**Background:**

Fulminant hepatic failure (FHF) is clinical syndrome with very poor prognosis and high mortality there is urgent need for the development of safe and non-toxic hepatoprotective agents for the adequate management of hepatitis. Hepatoprotective effect of the *Lepidium sativum* ethanolic extract (LSEE) was assessed by D-galactosamine-induced/lipopolysaccharide (400 mg/kg and 30 μg/kg) liver damage model in rats.

**Methods:**

Hepatoprotective activity of LSEE (150 and 300 mg/kg) and silymarin on D-GalN/LPS induced FHF in rat was assessed using several liver function enzyme parameters. Antioxidant properties as antioxidant stress enzymes were assessed in hepatic Liver as well as mRNA expression of cytokines genes such as TNF-α, IL-6, and IL-10 and stress related genes iNOS and HO-1 were determined by RT-PCR. Protein expression of apoptotic genes were evaluated through western blot. MPO and NF-κB DNA-binding activity was analyzed by ELISA. The magnitude of hepatic impairment was investigated through histopathological evaluation.

**Results:**

Marked amelioration of hepatic injuries by attenuation of serum and lipid peroxidation has been observed as comparable with silymarin (25 mg/kg p.o). D-GalN/LPS induced significant decrease in oxidative stress markers protein level, and albumin. LSEE significantly down-regulated the D-GalN/LPS induced pro-inflammatory cytokines TNFα and IL-6 mRNA expression in dose dependent fashion about 0.47 and 0.26 fold and up-regulates the IL-10 by 1.9 and 2.8 fold, respectively. While encourages hepatoprotective activity by down-regulating mRNA expression of iNOS and HO-1. MPO activity and NF-κB DNA-binding effect significantly increased and was mitigated by LSEE in a dose-dependent style as paralleled with silymarin.

**Conclusion:**

Our data suggests that pretreatment of LSEE down regulates the caspase 3 and up-regulates the BCl_2_ protein expression. The above findings revealed that *Lepidium sativum has* significant hepatoprotective activity.

**Electronic supplementary material:**

The online version of this article (doi:10.1186/s12906-016-1483-4) contains supplementary material, which is available to authorized users.

## Background

Fulminant hepatic failure (FHF), a severe disease with very low prognosis and high mortality is caused by bacteria, hepatitis virus, alcohol, toxic drugs and is characterized by massive hepatocytes necrosis [1]. There is lack of active therapies with high mortality (80–90%) detected in patients [2]. The general agreement among hematologist there is serious concern for finding out a range of effective medicine that can be described as hepatoprotective agents. However, various natural products of plant origin were studied for the mitigating effect in hepatic diseases such as phenols, lignans, coumarins, essential oils, glycosides, alkaloids, flavonoids, carotenoids etc. have been reported to possess mitigating hepatic effects [3–5]. Administration of single dose of D-GalN results in dose dependent hepatic damage resembling viral hepatitis, with focal necrosis and periportal inflammation. It induces hepatitis by hindering the synthesis of RNA and protein via reduction in cellular UTP uptake that tips to the hepatic parenchyma necrosis [6, 7]. The quinones, flavones and carotenoids are well known antioxidants that are claimed to be hepatoprotective were evaluated by D-GaLN/LPS hepatic damage model [8, 9].

Proinflammatory mediators comprising TNF-α, IL-6, IL-10, molecules play important roles [10, 11]. Oxidative stress causes increase of ROS by triggering of iNOS-2 [12]. HO-1, GPx1 and CAT are key enzymes, which play crucial function in shield against the oxidative stress prompted by toxins [13, 14]. Additionally, a proof exists NO is a major initiator of endotoxemia and inflammation [15]. TNF-α induced apoptosis and neutrophil transmigration, serve as a crucial mediator in the development of acute hepatic injury [16]. TNF-α is effective in patients with FHF. Furthermore, regarding the regulation of TNF-α, NF-κB plays crucial role situated in cytoplasm in inactive state then enters the nucleus which triggers transcription of TNF-α on LPS induction [17].

Natural medicinal products might offer a natural key to hepatoprotective effect against xenobiotic/drug [18]. *Lepidium sativum* L. (Brassicaceae) is medicinal plant origin to Egypt and Middle East, now cultivated in whole World. *Lepidium sativum* L used as remedy for inflammatory diseases, such as diabetes, arthritis, traumatic injuries, and hepatitis in traditional medicine [19, 20]. *Lepidium sativum* L. extract is reported to have various in vitro biological effects including antioxidant, anti-inflammatory, antidiarrheal, antimicrobial, antispasmodic and hepatoprotective action against oxidative damage and therefore, have a great potential for use as herbal hepatoprotective or dietary supplements [21, 22]. Literature on phytochemical investigations of *Lepidium sativum* L. revealed the presence of benzyl isothiocyanate, flavonoids, tannins, triterpens, alkaloids, sterols, glucosinolates [20, 23] which were reported to have, antioxidant, anti-inflammatory, analgesic activities and hepatoprotective properties [24–26]. The extensive literature survey showed that no mechanistic hepatoprotective studies had been carried out on this herb. The present research manuscript describes the Efficacy of *Lepidium sativum extract* in the amelioration of D-GalN/LPS induced liver injury and oxidative damage in animal model.

## Methods

### Plant materials and chemicals

*Lepidium sativum* L seeds were procured from a local market in Riyadh, Saudi Arabia. The seeds of *Lepidium sativum were* identified by Taxonomist in the college of pharmacy, King Saud University, Riyadh. A voucher specimen (32657) was deposited at the Herbarium of the College of Pharmacy, King Saud University, Riyadh, Saudi Arabia. The seeds were washed with double distilled water, de-shelled and dried and ground using pestle and mortar before extraction. The seeds were allowed to dry under the sunlight for two days. The seeds were then crushed and passed through a mesh #80 to get the fine powder and stored in dark containers free from moisture before experiments. Silymarin (99%) (Standard drug) and D-GalN/LPS (extracted from *Escherichia coli*) were purchased from Sigma chemicals. All chemicals and reagents used were of analytical grade and were purchased from Panrec Chemicals.

### Preparation of seed extracts

The extract of *Lepidium sativum* seeds was obtained by complete extraction using the Soxhlet extractor. 200 g of powdered seed sample was put into a porous thimble and placed in a Soxhlet extractor, using ethanol as extracting solvent for 8 h. The extract was obtained under reduced temperature, pressure and refluxing at 80 °C to remove the excess solvent from the extract. The extract was then stored in refrigerator for subsequent phytochemical analyses. The total yield obtained was 13.4% *w/w*.

### Phytochemical evaluation of LSEE

The preliminary screening of LSEE was carried out by gas chromatography-mass spectrometry (GC-MS) at the College of Pharmacy Research Center, King Saud University, Riyadh, Saudi Arabia, to study the phytochemical components present in LSEE. The extracts obtained from Soxhlet were filtered through Whatmann filter paper. The filtrate was concentrated by rotary evaporator (Büchi). The GC-MS analysis of extract was performed in a Perkin Elmer (USA), Clarus 600 gas chromatography linked to a mass spectrometer (Turbomass). The GC-MS conditions were kept same as per our previous report [27]. Briefly, an aliquot of 2 μL of extract was injected into the Elite -5MS column of 30 m, 0.25 mm film thickness, 0.25 μm internal diameters. Initial oven temperature of 30 °C for 2 min, increasing to 150 °C at a rate of 30 °C for 5 min and then again increases to 300 at the rate of 5 °C for 5 min. The injector temperature was maintained at 280 °C. The interface temperature was 220 °C and the electron energy used was 70ev. Helium was used as a mobile phase at a flow rate of 1.0 mL/min. Mass spectral detection was carried out in electron ionization mode by scanning at 40 to 600 (m/z). Finally, unknown compounds were identified by comparing the spectra with that of the National Institute of Standard and Technology library. The total time required for analyzing a single sample was 82 min.

### Hepatoprotective activity

#### Animals

Two month old Wistar male rats (180–205 g) were used for the investigation. Animals were kept at random and assigned to groups of 6 animals each in standard plastic animal cages with husk bedding. The animals were kept at 25 ± 2 °C with 12 h light and dark cycle. The rats were fed on standard pallet diet and provided free access of water ad libitum*.* Animals were maintained in accordance with the recommendations of the ‘Guide for the Care and Use of Laboratory Animals’ approved by institutional animal ethics committee of College of Pharmacy, King Saud University, Riyadh, Saudi Arabia (Clearance No. 05763–891; January 6, 2014).

### Acute toxicity

The acute oral toxicity study was conducted using the limit test procedure according to OECD test guidelines on acute oral toxicity test 401 [28]. The total of 6 rats of either sex were used in the investigation. LSEE was suspended in distilled water and 2 ml was administered to rats at a single dose (2000 mg/kg b.w) by gastric gavage. The rats were not fed for 3 h following administration. The rats were observed uninterruptedly for 1 h and then half hourly for 4 h for any major behavioral change and general motor signs like writhing, convulsion, response to tail pinching, gnawing, pupil size, fecal output, feeding activities, etc., and additionally up to 72 h for any mortality. The significant behavioral changes and mortality was observed (data not shown).

### Experimental procedure

Animals were arbitrarily divided into five groups of six animals each. Group I received normal saline for 14 days and served as normal control. Group II received normal saline (1 ml/kg, p.o.) for 14 days and served as toxic control. Groups III and IV were prophylactically treated with plant extract at a dose of 150 mg/kg p.o. and 300 mg/kg p.o each, respectively. Groups V served as positive control and were prophylactically treated with silymarin (50 mg/kg, p.o.) for 14 days. On 15^th^ day the groups II, III, IV and V, received D-GalN/LPS (400 mg/kg and 30 μg/kg, i.p.) [29]. After 24 h of D-GalN induced hepatotoxicity, the blood was withdrawn from retro orbital plexus under anesthesia in tubes containing di-sodium EDTA. Plasma was separated by centrifugation at 2500 × g for 10 min and was transferred to pre-labeled eppendrof tubes for various biochemical parameters. Straightaway, after blood withdrawal all the groups were sacrificed and liver tissue were collected for histopathological and biochemical assessments. Liver samples were washed with chilled normal saline, and 10% (*w/v*) liver homogenates were prepared in ice cold 0.15 M KCl solution using motor driven Teflon pestle.

### Determination of serum and liver homogenate biochemical parameters

Different biochemical parameters like ALT, AST [30, 31], ALP [32], γGGT [32]. Total protein [33], bilirubin and albumin, respectively were estimated in serum. TBARS, GSH [34], SOD [35], CAT [36], respectively were estimated in homogenized liver tissue. MPO level was evaluated by calculating the H_2_O_2_-dependent oxidation of o-dianizidine using MPO assay kit (ab105136) [37].

### Assessment of nitric oxide level

Nitric oxide was assessed by ratio of its stable metabolites, nitrite/nitrate. Nitric oxide levels were evaluated by Griess’ method [38].

### RNA isolation and quantification

Hepatic samples were used for total RNA isolation by Trizol reagent (Life Technologies). The total RNA was transcribed to cDNA by using high capacity cDNA reverse transcription kit of (Applied Biosystems). Quantitative analysis of cytokines and stress genes was performed by real time PCR by exposing the resulting cDNA to PCR amplification using 96-well optical reaction plates in the ABI Prism 7500 System (Applied Biosystems). Rat primers for TNF-α, IL-6, IL-10, HO-1, iNOS-2, and β-ACTIN gene [27] according to the manufacturer’s protocol. Fold variations paralleled to the controls were then evaluated by 2 − ΔΔCt. The fold change in the level of mRNA between treated and untreated groups were corrected by the levels of β-ACTIN. All reactions were run in duplicate.

### Preparation of nuclear and total protein extracts

Frozen hepatic tissue from different experimental groups was homogenized in ice-cold RIPA buffer comprising 1% protease inhibitor cocktail (Sigma-Aldrich) to get total protein extracts. After being centrifuged at 13,000 g for 20 min at 4 °C, the supernatant was collected and used for analysis of caspase-3, Bcl-2 and β-actin expression. Similarly, nuclear extracts were prepared by using NE-PER nuclear and cytoplasmic extraction kit (Pierce Biotechnology) containing 1% protease inhibitor cocktail (Sigma-Aldrich) according to the company’s protocol and used for analysis of NF-κB (p65) protein expression as well as NF-κB-DNA binding assay. The protein contents were measured by using lowery method against BSA as reference. Bands were visualized using Luminata™ Western Chemiluminescent HRP Substrates (Millipore, Billerica, MA, USA) and a densitometric analysis of the immunoblots was performed using LI-COR C-DiGit Blot Scanners (Lincoln, NE, USA).

### Analysis of NF-κB (p65) activation by ELISA

NF-κB DNA-binding activity was analyzed consuming the NF-κB(p65) transcription factor ELISA assay kit. (Cayman Chemical Company). Briefly, nuclear extracts were incubated in the oligonucleotide-coated wells where the oligonucleotide sequence contains the NF-κB response element consensus-binding site. After washing, samples were incubated by adding specific antibody directed against NF-κB (p65). HRP conjugated secondary antibody was added to give sensitive spectrometric readout at 450 nm.

### Histopathological studies

Hepatic tissues were removed instantly, sliced and washed in saline. Liver pieces were immersed in 10% formalin for histopathological studies. Tissue were processed and embedded in paraffin wax. Sections were taken and stained with hematoxylin and eosin and observed under microscope.

### Statistical data analysis

Results are expressed as mean ± SEM. Total variation present in a set of data was estimated by one-way analysis of variance (ANOVA) followed by Dunnet’^s^ *t*-test. *P < 0.01* was considered significant.

## Results

### Preliminary phytochemical analysis

The GC-MS analysis of *Lepidium sativum* extract revealed the presence of 48 phytochemical constituents that could contribute to the medicinal activity of the plant. Among the 48 phytochemical constituents identified in the extract are glucosinolates such as benzyl isothiocyanate, benzeneacetonitrile and phenolic content most prominent (Additional file 1 attached).

### Protective effect of LSEE on D-GalN/LPS induced hepatotoxicity

Preliminary studies on LSEE showed no toxicity in rat provided with dose up to 2000 mg/kg b.w. Therefore, for further studies 150 and 300 mg/kg b.w oral doses of extract have been selected (Additional file 2).

### Assessment of biochemical markers

A significant increase in serum biomarkers AST, ALT, ALP, γ-GT and bilirubin level was observed in animals administered with D-GalN/LPS, which is indicative of hepatic injuries. LSEE at a dose of 150 and 300 mg/kg p.o. pretreatment for 7 days reduced the levels of above mentioned parameters (*P* <0.05, *P* < 0.01, and *P* < 0.001) in groups III and IV. Moreover, silymarin (group V) pretreatment produced highly significant reduction (*P* < 0.001) in serum AST, ALT, ALP, γ-GT and bilirubin level. The amelioration of D-GalN/LPS induced hepatic injuries by LSEE is comparable with silymarin (Table 1).

**Table 1:** Effect of *Lepidium sativum* extract on liver and oxidative stress markers in D-GalN/LPS–induced hepatotoxicity

Serum	Control	D-GalN/LPS	Silymarin	D-GalN/LPS+ LSEE 150 mg/kg	D-GalN/LPS+ LSEE 300 mg/kg
AST U/L	75.80 ± 1.04	213.52 ± 2.59	93.95 ± 0.86	194.28 ± 1.32	126.25 ± 1.62
ALT U/L	33.94 ± 0.98	95.96 ± 1.21	52.39 ± 1.20	82.93 ± 1.61	60.96 ± 1.56
ALP U/L	81.09 ± 1.80	180.86 ± 3.38	94.07 ± 2.03	150.10 ± 2.58	103.76 ± 1.30
Bilurubine plasma mg/dL	0.72 ± 0.01	1.01 ± 0.01	0.69 ± 0.01	0.92 ± 0.012	0.70 ± 0.01
γ-GGT	1.26 ± 0.06	3.12 ± 0.14	1.70 ± 0.04	2.49 ± 0.04	2.01 ± 0.08
CAT U/mg	45.09 ± 1.07	20.98 ± 1.06	39.63 ± 1.63	24.43 ± 1.22	34.18 ± 1.01
GSH nmol/mg	1.17 ± 0.02	0.46 ± 0.02	0.98 ± 0.02	0.71 ± 0.01	0.86 ± 0.02
MDA nmol/minute/mg	3.20 ± 0.10	9.80 ± 0.23	3.95 ± 0.16	7.60 ± 0.18	6.36 ± 0.14
SOD U/mg protein	22.24 ± 0.41	10.53 ± 0.35	20.49 ± 0.85	17.35 ± 0.60	19.57 ± 0.54

Data presented as mean ± SEM

### Assessment of oxidative stress

Role of LSEE on the Level of GSH, SOD, CAT and D-GalN/LPS intoxicated rats (group II) showed a significant reduction of GSH, SOD, CAT and total protein content indicative of an increase in protein metabolism whereas there was a dose dependent increase in GSH, SOD, CAT and total protein content significantly in groups III and IV (LSEE 150 and 300 mg/kg + D-GalN/LPS). Silymarin pretreatment (group V) produced highly significant increase in GSH (*P* < 0.001) (Table 1).

### Assessment of antioxidant activity

The degree of lipid peroxidation is assessed by the formation of thiobarbituric acid reactive substances (TBARS). There was a sharp increase in TBARS level in D-GalN/LPS intoxicated rats (group II) indicative of oxidative stress. The LSEE 150 and 300 mg/kg + D-GalN/LPS intoxicated rats (groups III and IV) showed significant dose dependent reduction of TBARS level as compared to D-GalN/LPS -treated rats (group II). Table 1 displayed significant change in the antioxidant contents of TBARS in D-GalN/LPS intoxicated rats as 6.92 ± 0.93 (*P*<0.001) compared to control group. The change in TBARS level is comparable with silymarin.

### Assessment of LSEE on mRNA expression of cytokines genes and stress gene

**Fig. 1:**
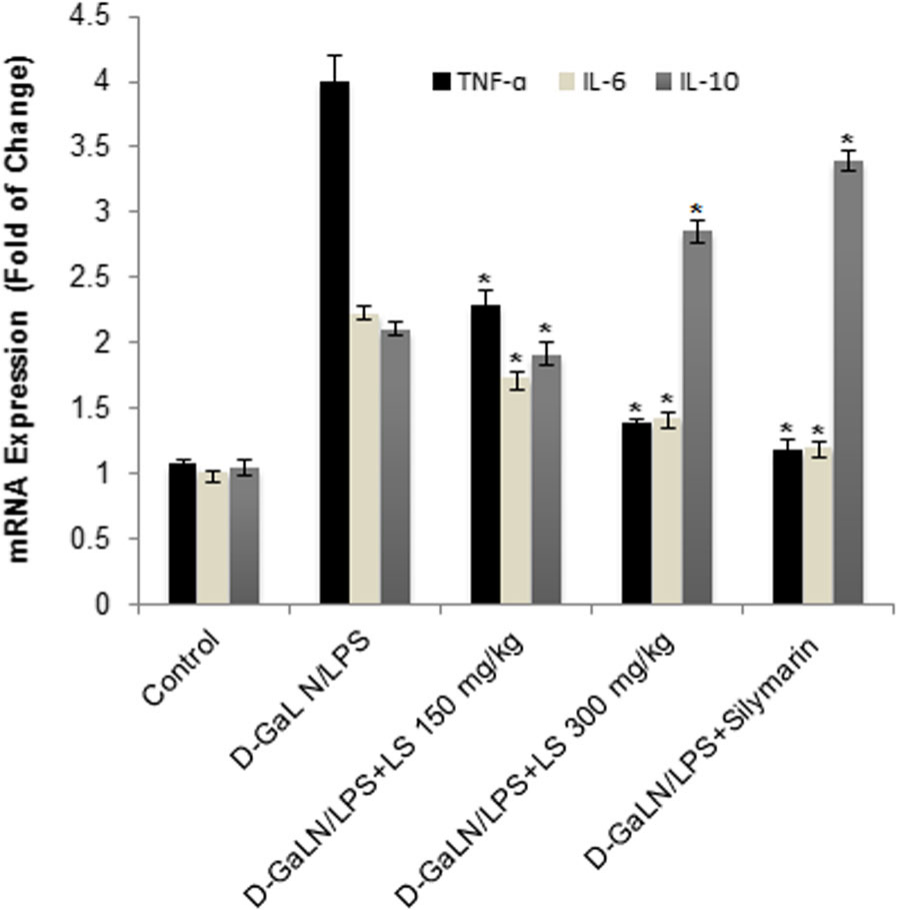
Effect of Lepidium sativum extract on mRNA expression of cytokines genes such tumor necrotic factor-α (TNFα), Interluekine 6 (IL-6), and Interluekine 10 (IL-10). All values represent mean ± SEM. *p < 0.05; ANOVA, followed by Dunnett’s multiple comparision test. *compared to GalN/LPS only group

The hepatic level of TNF-α and IL-6 on mRNA expression in D-GalN/LPS intoxicated rats was up-regulated approximately 3.77 and 2.2 fold higher as comparable to normal rats (Fig. 1). In animals receiving standard drug silymarin, hepatic expression of TNF-α and IL-6 were down regulated by 0.29 and 0.54 fold as compare to D-GalN/LPS intoxicated rats. Interestingly LSEE down regulate the hepatic TNF α and IL-6 mRNA expression significantly in dose dependent fashion as compare to D-GalN/LPS intoxicated rats. In addition, treatment of rats with LSEE for one week prior to D-GalN/LPS administration enhanced the mRNA expression of IL-10, as shown in Fig. 1, which adds more proof of the anti-inflammatory effect of LSEE in the acute phase response to D-GalN/LPS.

**Fig. 2:**
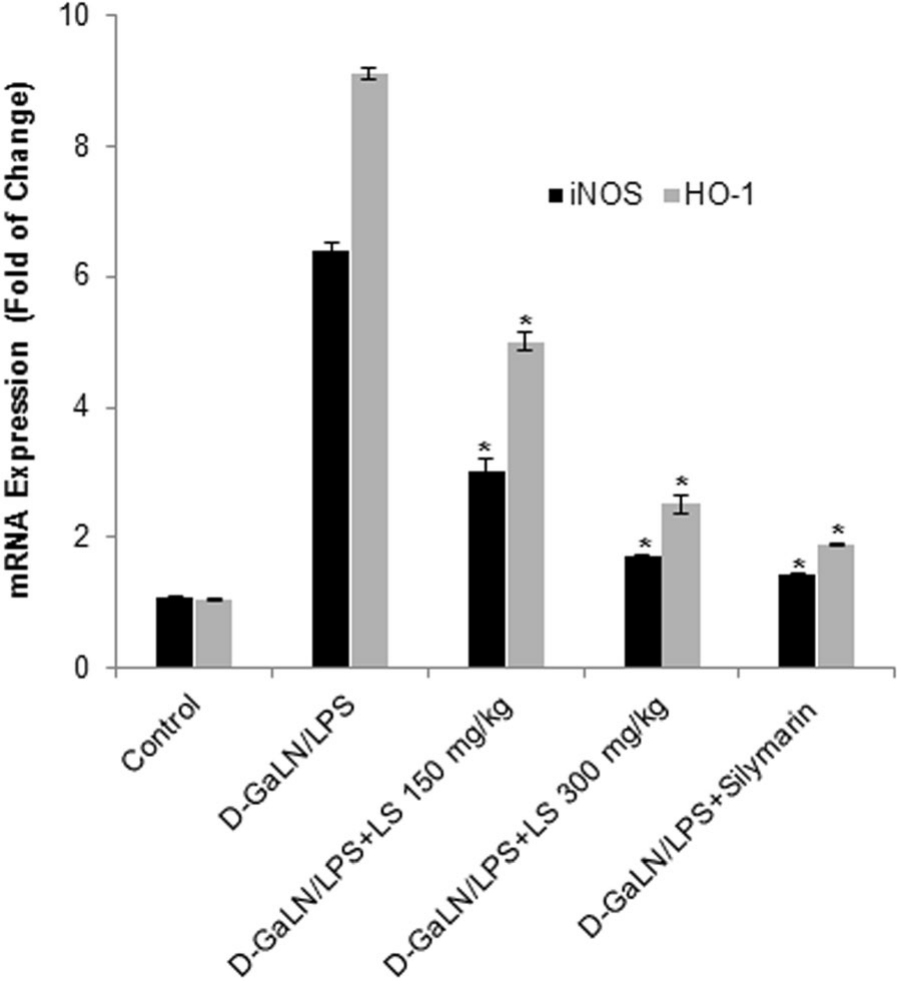
Effect of Lepidium sativum extract on mRNA expression of cytokines genes such nitrous oxide synthase (iNOS) and Haemoxygensae1 (HO-1). All values represent mean ± SEM. *p < 0.05; ANOVA, followed by Dunnett’s multiple comparision test. * compared to GalN/LPS only group

The up-regulation of IL-10 mRNA by LS EE indicates its ability to down-regulate the inflammatory cytokine. The hepatic level of iNOS-2 on mRNA expression in D-GalN/LPS intoxicated rats was approximately 6.04-fold higher than that of normal rats. There is a dose dependent significant down-regulation in rat groups previously treated with LS EE (150 and 300 mg/kg), silymarin + D-GalN/LPS (Fig. 2). This is about 0.47 and 0.26 down regulation in LSEE+ D-GalN/LPS rats. While silymarin and D-GalN/LPS rats showed 0.22 fold down regulation of iNOS2 mRNA in rat’s liver and this closely correlates with nitrites levels measured in various groups (Fig. 2). This further indicates that LSEE and silymarin have anti-inflammatory activities. The degree of heme catabolism, as shown in Table 1, illustrated considerably high levels of bilirubin in serum of D-GalN/LPS -intoxicated rats as compared to the control group. The same inclination is witnessed in the inducible HO-1 on mRNA expressions which were significantly increased 8.9 -fold (*P* ≤ 0.01) in D-GalN/LPS group compared to control (Fig. 2). On the other hand, control did not exercise any significant changes in HO-1 on mRNA expressions LSEE and silymarin significantly (*P* ≤0.01) down regulate the levels of HO-1 on mRNA expression by 4.87, 2.45, and 1.8 fold in groups III, IV, and V, respectively.

### Assessment of MPO Level

**Fig. 3:**
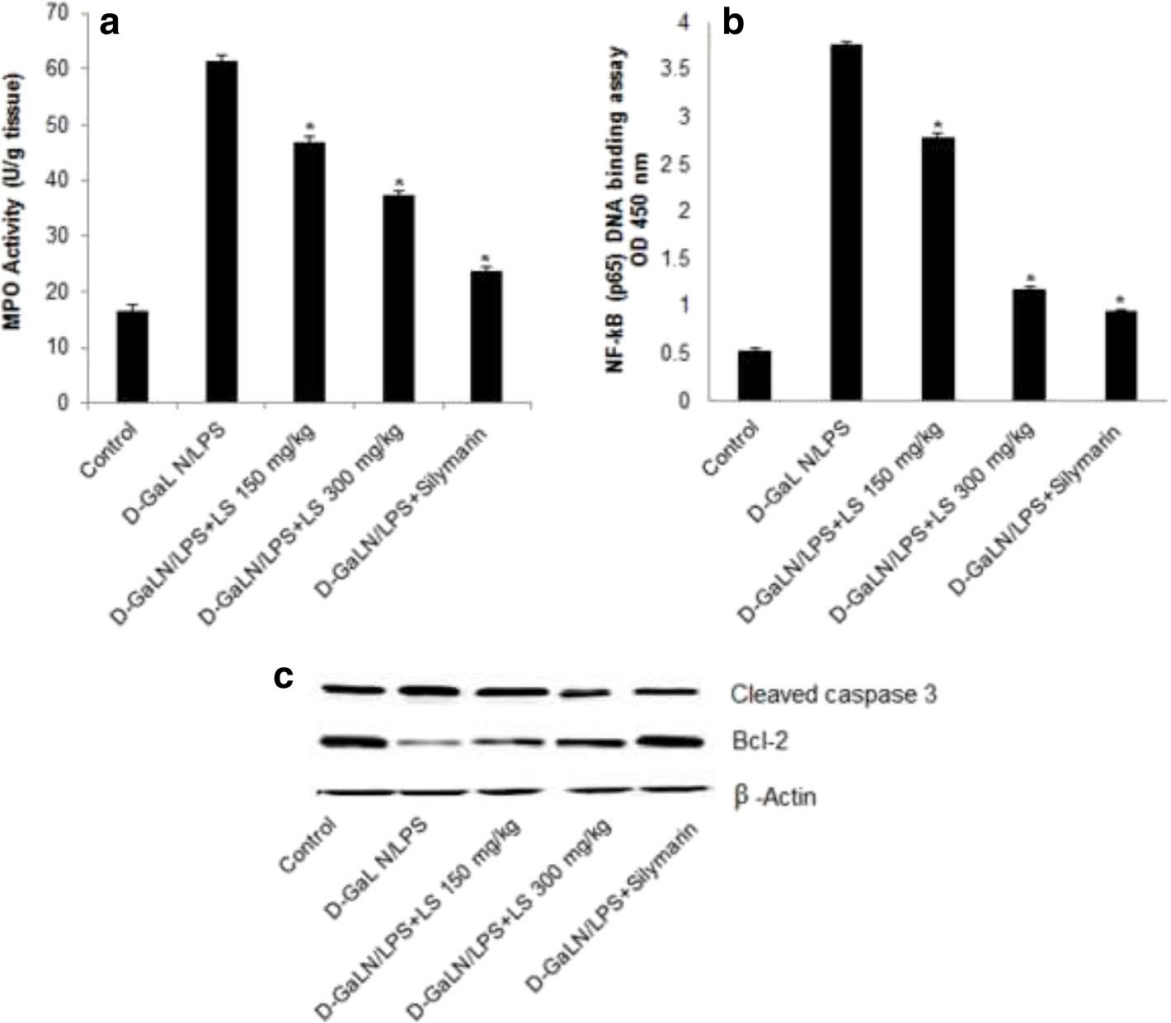
Effect of LSEE on D-GalN/LPS-induced changes in inflammatory and apoptotic markers in Liver tissues of rats. **a** Myeloperoxidase (MPO) (**b**) Nuclear NF-κB (p65) DNA-binding activity determined by using NF-κB (p65) transcription factor ELISA assay kit. **c** Immunoblot analysis of apoptotic marker cleaved caspase-3 and antiapoptotic marker Bcl-2 protein in comparison with β-actin expression was used as a loading control. All values represent mean ± SEM. *p < 0.05; ANOVA, followed by Dunnett’s multiple comparision test. * compared to GalN/LPS only group

MPO content considerably increased after D-GalN/LPS treatment and this boost was mitigated by LSEE in a dose-dependent fashion. MPO content is comparable with silymarin Fig. 3a.

### Assessment of LSEE D-GalN/LPS induced the activity of NF-κB

The nuclear NF-κB level was assessed by using a NF-κB p65 transcription factor assay method. As displayed in Fig. 3b, in D-GalN/LPS group, NF-κB activity distinctly enhanced compared with the control group. However, LSEE and silymarin pretreatment vividly inhibited D-GalN/LPS -induced NF-κB activity in a dose-dependent fashion.

### Effect of LSEE on caspase-3, and bcl-2 expression

The caspase-3, and bcl-2 protein expression was detected 12 h after D-GalN/LPS administration. There is up regulation of caspase-3 and down regulation of bcl-2 expression after D-GalN/LPS administration. LSEE treatment down regulates the caspase-3 while up regulate the bcl-2 expression about 1.7 fold as compared to the control group (Fig. 3c).

### Histopathological observations

**Fig. 4:**
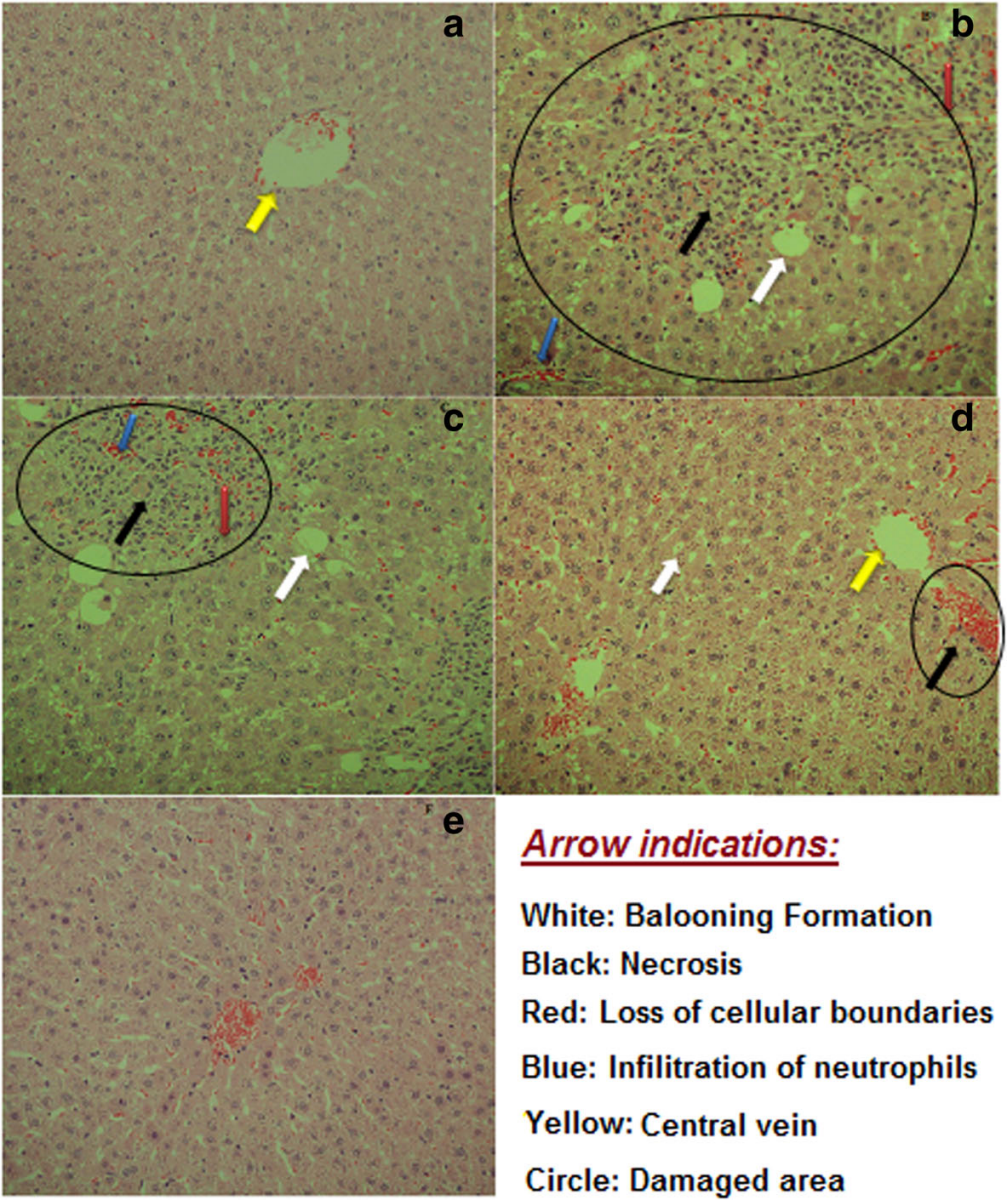
Histopathology of liver tissues. **a** Liver section of normal control rat shows central vein surrounded by hepatic cord of cells (normal architecture), **b** liver section of D-GalN/LPS treated rats showing massive fatty changes, focal central vein congestion, ballooning formation, necrosis with inflammation and loss of cellular boundaries, **c** liver section of rats treated D-GalN/LPS and 150 mg/kg of LSEE showing mild central vein congestion (indicated by arrow), ballooning, necrosis with sinusoidal dilatation, **d** liver section of rats treated D-GalN/LPS and 300 mg/kg of LSEE showing absence of ballooning, inflammatory cells and regeneration of hepatocytes around central vein toward near normal liver architecture but slight congestion in central vein (indicated by arrow), **e** liver section of rats treated D-GalN/LPS and 10 mg/kg of silymarin showing normal liver architecture

Findings of histopathological changes in liver due to d-GalN/LPS are shown in Fig. 4. Histology normal control animals liver (Group I) displayed normal Browicz-Kupffer cells architecture with well-defined central vein, well preserved cytoplasm with prominent nucleus (Fig. 4a). The D-GalN/LPS treated animals (Group II) displayed Browicz-Kupffer cells with severe toxicity described by scattered inflammation across liver parenchyma, necrosis around the vein, enlargement up of vascular endothelial cells and inflammatory cell collection (Fig. 4b). Moderate to high hepatic protection from d-GalN/LPS was achieved in *Lepidium sativum* with dose of 300 mg/kg. This group showed improved liver structure with minimal cellular necrosis. Inflammatory changes inflicted by D-GalN/LPS were remarkably reversed by treatment with both doses of *Lepidium sativum* 150 and 300 mg/kg. They seemed to significantly inhibit the d-GalN/LPS toxicity as shown by the Browicz-Kupffer cells with well-preserved cytoplasm as shown in Fig. 4c and d. Silymarin (Group V) pre-treatment showed normalization of fatty changes, cellular infiltration and necrosis to highly significantly prevention of d-GalN/LPS toxicity as shown by the Browicz-Kupffer cells with well-preserved cytoplasm (Fig. 4e). Furthermore, the histological evaluation of liver tissues showed that D-GalN/LPS caused massive fatty changes, focal central vein congestion, and necrosis with inflammation, ballooning formation and loss of cellular boundaries. *Lepidium sativum* seed extract treatment improved liver histology towards more normalization especially with the 300 mg/kg dose.

## Discussion

Hepatic damages inflicted by D-GalN/LPS are the well described method of xenobiotic induced hepatotoxicity and frequently employed models for the selection of anti-hepatotoxic or hepatoprotective agent. There is serious necessity for the development of safe and secure hepatoprotective agent of plant origin for the management of hepatitis. Natural herbs which hold anti-inflammatory and antioxidant activity has become a main focus for exploration to mitigate tissue damages. The D-GalN/LPS inflicted hepatic injuries are parallel to that of viral hepatitis [39, 40]. Therefore, D-GalN/LPS assisted hepatic damage was designated as the experimental model. D-GalN/LPS alter the antioxidant activity of organs, vulnerable to oxidative stress. Previous reports have revealed that D-galactosamine causes shift of liver biomarker enzymes [41]. D-GalN/LPS is recognized to inhibit the transcription and translation as a result of endotoxemia, it causes fulminant hepatitis [42]. D-GalN membrane architecture is believed to be accountable for the impairment of ionic pumps. The damage in the calcium pumps, with subsequent upsurge in the calcium leading to cell sense [40]. D-GalN/LPS administrations may lead to marked elevation in hepatic markers [40].

In the present research the rise in AST, ALT, ALP, γ GGT and bilirubin levels encouraged by D-GalN treatment was significantly abridged by LSEE (150 and 300 mg/kg) and silymarin *pre*-treatment ameliorate the hepatic injuries might be due its consequence against impairment of ionic pumps, cell leakage and loss of membrane integrity. D-GalN/LPS may alter the antioxidant capacity of the organs [43]. The administration LSEE to D-GalN/LPS intoxicated rats increases the hepatic antioxidants effect and reduces the oxidative stress, described reduced TBARS concentration and increase in GSH, SOD and CAT contents. GSH is a strong antioxidant that shields cells from the oxidative damages and fall in GSH pool can make cells venerable to oxidative stress [44]. In addition, GSH and CAT work simultaneously to counteract the oxidation of proteins, lipids and DNA by abolishing ROS [45]. LSEE exhibits DPPH- scavenging activity, which supplementary endorses its antioxidant activity. D-GalN is described to inflammatory response in liver.

The D-GalN/LPS displayed severe necrosis and inflammation in hepatic parenchymal cells and around portal track. The LSEE pretreatment attenuates the myeloperoxidase (MPO) level in dose dependent manner these effects showed that LSEE has ability to down-regulated the inflammation to shield against D-GalN/LPS-inflicted hepatic injuries. Similar effects were observed in silymarin treated animals. Several antioxidants may possess anti-inflammatory activity [46], therefore, evaluation of in vivo anti-inflammatory activity of LSEE on cytokines genes TNF-α and IL-6 mRNA expression was down regulated in D-GalN/LPS intoxicated rats. TNF-α has a key role in the pathogenesis of D-GalN/LPS-induced FHF and apoptosis in liver [47]. In fact, TNF-α acts by caspase-8 and then stimulates caspase-3, a downstream cysteine proteinase via various apoptotic pathways [47]. Interestingly, *LSEE* treatment markedly reduced TNF-α and IL-6 mRNA expression, and at the same time, enhanced the level of IL-10. Additionally, The liver involving oxidative stress switch the up-regulation of HO-1 mRNA expression and upsurge in products of heme degradation pathway [46–48]. Heme deprivation lead to production of biliverdin/bilirubin and carbon monoxide, the crucial mediators of inducible HO-1 arbitrated cellular protection, that is why they restore homeostatic balance and diminishing inflammation via down regulation of cytokines [48, 49]. The results of HO-1 gene expression data showed that D-GalN/LPS increases HO-1 level, while pretreatment with *LSEE* D-GalN/LPS rats exhibited a marked reduction in HO-1 mRNA expression. This clearly indicates that *LSEE* has a cellular protection role as well. The iNOS 2 expression and nitrite contents were significantly augmented following D-GalN/LPS administration, that can be ascribed to the stated D-GalN/LPS -prompted NO production [50]. Similarly, *LSEE* pretreatment had a significantly down regulate iNOS-2 expression and nitrate content in D-GalN/LPS intoxicated rats furthermore, this testified that isothiocyanates arbitrates its cellular protection by down-regulating NO production owing to induction of HO-1 [51].

LSEE treatment significantly attenuate nitric oxide induced oxidative stress progressed to reduction in inflammation as well as vasoconstriction of endothelial cells [52]. The activation and nuclear translocation of NF-κB, in response to oxidative stress/nitrosative stress, are the key factors in the hepatic inflammatory process by regulating the gene expression of cytokines, chemokine’s, and adhesion molecules [53, 54]. This investigation, LSEE (150–300 mg/kg) pretreatment significantly down regulate the nuclear NF-κB expression and NF-κBDNA binding activities as well as cytokines (TNF-α and IL-6) in dose dependent fashion as compared to D-GalN/LPS intoxicated rats. Our findings corroborate those of earlier studies demonstrating that an up surge in NF-κB activation is also followed by an increase in the concentration of inflammatory cytokines like TNF-α and IL-6. Apoptosis is a noticeable feature of hepatic disease.

D-GalN/LPS liver damage model exhibited the apoptosis [11] and [55]. The apoptotic and anti-apoptotic proteins such as Bcl-2 family play key role in regulation of apoptosis. The apoptotic hepatic damage in reaction to d-GalN/LPS is largely TNF-α dependent [47]. Therefore, it is expected that LSEE administration reduced the apoptosis. We have found increase caspase 3 and decrease Bcl-2 protein expression in d-GalN/LPS intoxicated rats leading to selective depletion of UDP in hepatic cells. The pretreatment of LSEE significantly down regulate the Caspase 3 and up regulate Bcl-2 protein expression in D-GalN/LPS inflicted liver leading to suppression of apoptosis. These outcomes corroborate previous investigations [47, 56, 57]. The probable activity of LSEE may be due to the presence of secondary metabolites as revealed by the GC MS data the presence 48 metabolite such as glucosinolates (benzyl isothiocyanate, benzene acetonitrile, benzo1-(2-benzo[b] thieny), Flavonoids (caryophyllene, 2,12-epoxycaryophyll-5), tannins, triterpens (Pseduosarsasapogenin-5,20-dien), alkaloids, sterols (ergosta-5,22-dien-3-ol, acetate, 3-chloro-5-cholestene, cholesta-8,24-dien-3-ol). These results were similar to previous reports [20, 23]. Which are known to have antimicrobial, antioxidant, antinflamatory and hepatoprotective properties in other cruciferous plants? The glucosinolates also known as sulforaphane exerts its effect by raising GSH (NP-SH) which indirectly by stimulating the Antioxidant Response Elements (ARE) in the 5′-upstream region of the gene for the heavy subunit of γ-glutamylcysteine synthetase [58]. Sulphophranes induced HO-1 and produce bilirubin a potent antioxidant [59]. Flavonoids such as caryophyllene a potent antioxidant, antinflamatory and anti-apoptotic agent by blocking ROS mediated MAPK activation [60]. Hexadecanoic acid may induced apoptosis and down regulation cytokines such TNFα, IL-6 [61]. Natural sterols such ergosterol peroxide significantly blocked MyD88 and VCAM-1 expression, and cytokine (IL-1β, IL-6 and TNF-α) production in LPS-stimulated cells. It also effectively inhibited NF-kB activation [62]. The possible activity of LSEE may be due to the presence of glucosinolates, flavonoids and natural sterols. These findings established that LSEE mitigates hepatic injuries and structural damage through the decline of oxidative stress, inflammation, and apoptosis in the D-GalN/LPS induced liver damage.

## Conclusion

Findings of this study revealed that LSEE is effective in prevention of d-GalN/LPS induced hepatic damage in rats. The pretreatment with LSEE significantly prevented the d-GalN/LPS induced up surge in liver function enzymes (AST, ALT, γ-GGT, ALP, total bilirubin, LDH, and Total protein). Thus significantly mitigate the reduction of lipid peroxidation and restored the antioxidant enzymes and total protein to normal levels. The hepatoprotective consequence of LSEE is attributed to down regulation cytokines (TNF-α, IL-6) and stress gene (iNOS and HO-1) mRNA expression and up regulation IL-10. Nuclear NF-κB (p65), NF-κB-DNA binding activity, myeloperoxidase (MPO) activity, and nitric oxide level were significantly down regulated upon LSEE pretreatment. In addition, The LSEE pretreatment significantly down regulate the apoptotic proteins such as Caspase 3 however up regulate Bcl 2 protein expression in D-GalN/LPS inflicted liver preventing apoptosis. LSEE pretreatment also ameliorated the degree of structural damage and abridged inflammatory infiltration in hepatic cells. These findings established that LSEE mitigates hepatic injuries and structural damage through the decline of oxidative stress, inflammation, and apoptosis in the liver.

## Additional file

Additional file 1: Supplementary data file 1. (DOCX 64 kb)
Additional file 2: Supplementary data file 2. (DOCX 12 kb)

## Abbreviations

ALP: Alkaline Phosphatase
ALT: Alanine aminotransferase
ARE: Antioxidant Response Elements
AST: Aspartate Aminotransferase
CAT: Catalase
D-GalN/LPS: D-galactosamine-induced/lipopolysaccharide
FHF: Fulminant hepatic failure
GC-MS: Gas chromatography-mass spectrometry ()
GSH: Glutathione
HO-1: Heme Oxygenase-1
IL-10: Interleukine-10
IL-6: Interleukine-6
iNOS: Inhibitory nitric oxide synthase
LSEE: *Lepidium sativum* ethanolic extract
MPO: Myeloperoxidase
NF-κB: Nuclear factor kappa B
ROS: Reactive oxygen species
SOD: Superoxide dismutase
TBARS: Thiobarbituric acid reactive substances
TNF-α: Tumor Necrosis Factor-α
γGGT: γ-glutamyl transpeptidase
