# Supplementary material for: Hepatoprotective activity of Lepidium sativum seeds against D-galactosamine/lipopolysaccharide induced hepatotoxicity in animal model
Source: BMC Complement Altern Med. 2016 Dec 3;16:501. doi: 10.1186/s12906-016-1483-4 (PMC5135812; doi:10.1186/s12906-016-1483-4)
Supplement: Additional file 1: — Supplementary data file 1. (DOCX 64 kb) [file 12906_2016_1483_MOESM1_ESM.docx]

**SUPPLIMENTARY DATA File 1 FOR REVIEW ONLY:**

**Preliminary phytochemical analysis of *Lepidium sativum* extract by GC-MS revealed the presence of 48 phytochemical constituents that could contribute to the medicinal activity of the plant.**


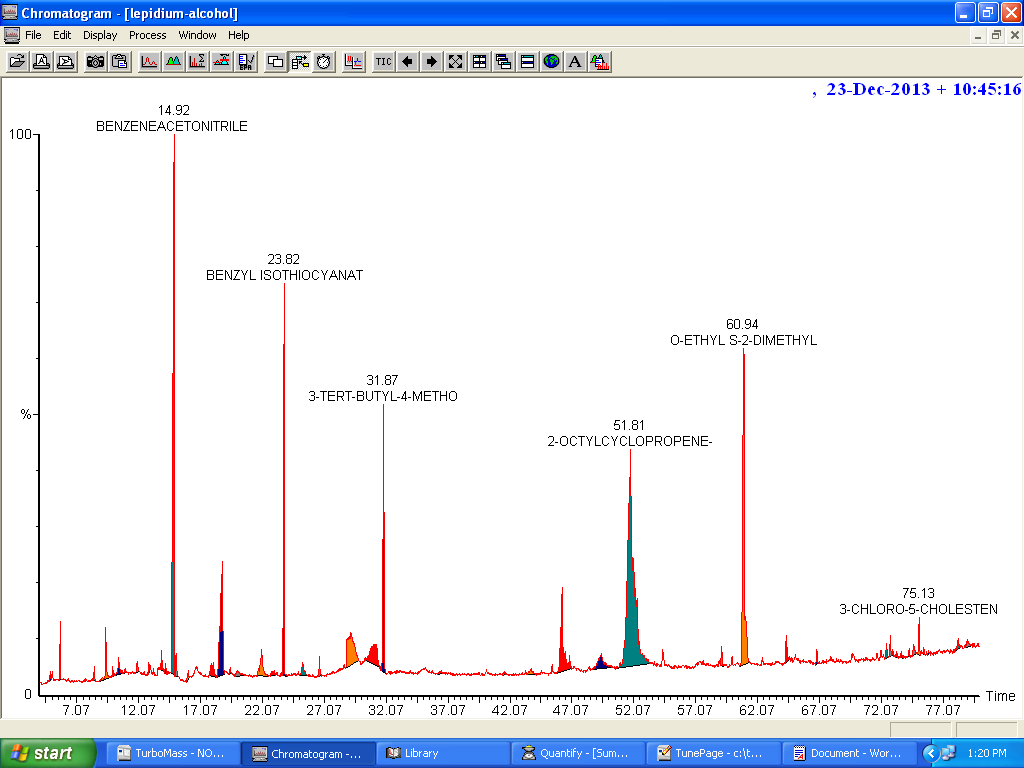


GC-MS Chromatogram of LSEE

**SECONDARY Metabolites:**

Sample Name: lepidium-alcohol Sample ID: S R AHAMAD

# Name RT Area Height N Area % Area %

1 2-PYRROLIDINECARBOXAMIDE, 5-OX 4.92 30214 501203 0.790 0.240

2 2-FURANCARBOXALDEHYDE (CAS) $$ 5.71 150063 3411472 3.930 1.170

3 2,4-PENTANEDIOL, 2-METHYL- (CA 8.46 35901 651040 0.940 0.280

4 BENZALDEHYDE (CAS) $$ PHENYLME 9.40 87646 1491812 2.290 0.680

5 2,3-DIHYDRO-3,5-DIHYDROXY-6-ME 9.96 27784 438114 0.730 0.220

6 N,N-DI-(N-BUTYL)-2-PYRIDYLETHY 10.44 61594 592293 1.610 0.480

7 BENZENEETHANAMINE, 3-BENZYLOXY 10.95 11075 242911 0.290 0.090

8 3-AMINO-2-OXAZOLIDINONE 11.97 76000 551595 1.990 0.590

9 METHANEDIAMINE, N,N,N',N'-TETR 12.88 28327 446035 0.740 0.220

10 LEVOGLUCOSENONE 13.90 43022 486173 1.130 0.340

11 BENZENEMETHANOL, .ALPHA.-[(ETH 14.24 9177 176927 0.240 0.070

12 BENZENEACETONITRILE (CAS) $$ B 14.92 2240773 17881466 58.670 17.450

13 2,3-DIHYDRO-3,5-DIHYDROXY-6-ME 15.09 95744 975054 2.510 0.750

14 2-BUTENETHIOIC ACID, 3-(ETHYLT 16.05 9263 156552 0.240 0.070

15 1H-INDENE, 1-CHLORO-2,3-DIHYDR 18.12 36717 579854 0.960 0.290

16 2-FURANCARBOXALDEHYDE, 5-(HYDR 18.83 668975 4143360 17.520 5.210

17 2,3-ANHYDRO-D-GALACTOSAN 19.45 57448 267760 1.500 0.450

18 2,4-DIMETHYL-3-TOSYL-2,5-DIHYD 20.02 54883 214607 1.440 0.430

19 3-PYRROLIDINOL 21.98 81046 408301 2.120 0.630

20 N-(2-METHOXYETHYL)ALANINE 23.00 16305 85849 0.430 0.130

21 BENZYL ISOTHIOCYANATE $$ BENZE 23.82 1706752 21155824 44.690 13.290

22 BENZENEACETAMIDE (CAS) $$ 2-PH 25.26 65750 404497 1.720 0.510

23 2H-1-BENZOPYRAN-2-ONE (CAS) $$ 26.68 46038 646782 1.210 0.360

24 BENZOIC ACID, 2-(DIMETHYLAMINO 29.06 988342 1960670 25.880 7.700

25 D-ALLOSE 31.18 335741 944277 8.790 2.610

26 3-TERT-BUTYL-4-METHOXYPHENOL $ 31.86 498844 5519435 13.060 3.880

27 3-ACETYL-3"'-PHENYL[4]STAFFANE 36.77 1048 36981 0.030 0.010

28 2-OCTEN-1-OL (CAS) $$ 2-OCTENO 41.64 4557 75004 0.120 0.040

29 PRILOCAINE $$ PROPITOCAINE $$ 43.77 34750 278495 0.910 0.270

30 HEXADECANOIC ACID (CAS) $$ PAL 46.29 256003 1303506 6.700 1.990

31 1-(2-BENZO[B]THIENYL)-TRANS-2, 49.29 31659 146873 0.830 0.250

32 2-OCTYLCYCLOPROPENE-1-HEPTANOL 51.84 660195 2354792 17.290 5.140

33 N,N-DIMETHYLDODECANAMIDE 59.18 37985 396356 0.990 0.300

34 O-ETHYL S-2-DIMETHYLAMINOETHYL 60.99 3819389 23317412 100.000 29.740

35 (2S,4S,6S,8R,9S)-4-(DIMETHYL-T 64.39 22809 420304 0.600 0.180

36 2-TRIDECEN-1-OL 66.86 35555 276591 0.930 0.280

37 4-HYDROXYTETRADEC-2-YNAL $$ 2- 72.50 42027 343843 1.100 0.330

38 3-CHLORO-5-CHOLESTENE 72.81 39365 454178 1.030 0.310

39 CHOLESTA-8,24-DIEN-3-OL, 4-MET 72.98 13665 266976 0.360 0.110

40 (2R,5E)-2,12-EPOXYCARYOPHYLL-5 73.26 68108 248064 1.780 0.530

41 CARYOPHYLLENE DIEPOXIDE 73.69 9718 119408 0.250 0.080

42 CHOLESTA-8,24-DIEN-3-OL, 4-MET 74.31 56382 285104 1.480 0.440

43 4-(5',5'-DIMETHYL-2'-METHYLIDE 74.71 38235 227025 1.000 0.300

44 3-CHLORO-5-CHOLESTENE 75.13 63857 746774 1.670 0.500

45 CHOLEST-5-EN-3-OL (3.BETA.)- ( 75.53 18228 167837 0.480 0.140

46 1(10)E,5E-GERMACRADIEN-4-OL 76.04 33785 224128 0.880 0.260

47 PSEDUOSARSASAPOGENIN-5,20-DIEN 78.25 39267 245237 1.030 0.310

48 ERGOSTA-5,22-DIEN-3-OL, ACETAT 79.02 50674 334016 1.330 0.390
