# Supplementary material for: Hepatoprotective activity of Lepidium sativum seeds against D-galactosamine/lipopolysaccharide induced hepatotoxicity in animal model
Source: BMC Complement Altern Med. 2016 Dec 3;16:501. doi: 10.1186/s12906-016-1483-4 (PMC5135812; doi:10.1186/s12906-016-1483-4)
Supplement: Additional file 2: — Supplementary data file 2. (DOCX 12 kb) [file 12906_2016_1483_MOESM2_ESM.docx]

**SUPPLIMENTARY DATA file 2 FOR REVIEW ONLY:**

General Screening of *Lepidium sativum* extract 2000 mg/kg b.w in either sex rats (n=6).

| S.No. | Toxic Symptoms Under Observation | General Screening of LSEE rats (n=6) of either sex |
| --- | --- | --- |
|  |  | 2000 |
| I | AUTONOMIC RESPONSES |  |
| 1 | Respiration | + |
| 2 | Hypothermia | + |
| 3 | Hyperthermia | - |
| 4 | Heart rate | - |
| 5 | Pilo erection | - |
| 6 | Salivation | - |
| 7 | Micturation | + |
| 8 | Defecation | ++ |
| 9 | Writhing | + |
| II | MOTOR ACTIVITY |  |
| 1 | Staggering | - |
| 2 | Reflex impairment | - |
| III | CNS EXCITATION | - |
| 1 | Straub tail | - |
| 2 | Tremors | + |
| 3 | Convulsions | + |
| 4 | Twitches | - |
| 5 | Aggression | + |
| 6 | Excitation | - |
| 7 | Itching | - |
| 8 | Waltzing movement | - |
| 9 | Muscle tone | - |
| 10 | Ocular | - |
| 11 | Dermal | - |
| IV | SYMPTOMS OTHER THAN ABOVE |  |
| 1 | Sedation | + |
| 2 | Calmness | - |
| 3 | Mortality(Dead/Total) | 0/6 |
